# Risk Factors for Radiologic Subaxial Cervical Pathology After C1-2 Posterior Fusion

**DOI:** 10.3390/jcm15051852

**Published:** 2026-02-28

**Authors:** Chungwon Bang, Kee-won Rhyu, Young-Yul Kim, Joonghyun Ahn, Ji-hyun Ryu, Hyung-Youl Park, You Seung Chun, Kihyun Kwon, Sang-Il Kim, Hyoung Ju Seo, Young-Hoon Kim

**Affiliations:** Department of Orthopedic Surgery, Incheon St. Mary’s Hospital, College of Medicine, The Catholic University of Korea, Seoul 03083, Republic of Korea; freeboy3645@gmail.com (C.B.);

**Keywords:** C1-2 fusion, subaxial cervical spine, rheumatoid arthritis

## Abstract

**Background/Objectives**: Atlantoaxial posterior fusion has unique characteristics, and it is anticipated that adjacent segment degenerative changes following fusion surgery may present distinctive findings. This study aims to analyze the risk factors for degenerative changes in subaxial levels following the increasingly common atlantoaxial posterior fusion procedure. **Methods**: A total of 58 patients (19 males, 39 females) who had neutral, flexion, and extension plain lateral radiographs taken and a follow-up record of approximately two years post-surgery were included in the final study cohort. The study analyzed surgical methods, patient demographics, hospitalization-related factors, visual analog scale (VAS) for neck pain, and radiologic parameters. Patients were classified into the radiologic subaxial pathology (RSP) group (*n* = 34) and the non-RSP group (*n* = 24) using several radiologic indicators of spinal instability or arthritic changes, and the risk factors for RSP were analyzed. **Results**: The RSP group showed a significantly higher proportion of females and prevalence of rheumatoid arthritis (RA). At 3 months postoperatively, the C1-7 sagittal vertical axis (SVA) was significantly lower in the RSP group. Multivariate regression analysis using significant variables (*p* < 0.05) such as sex, RA and 3-month C1-7 SVA showed that RA and 3-month C1-7 SVA were significantly associated with RSP. Among radiologic parameters related to surgery, multivariate analysis identified 3-month C1-7 SVA as the sole risk factor for RSP. To explore its correlation with other radiologic parameters at 3 months postoperatively, linear logistic regression analysis was conducted. Significant positive correlations were observed with the C1-2 Cobb angle. **Conclusions**: This study identified RA and C1-7 SVA as the most significant risk factors for RSP in atlantoaixal posterior fusion.

## 1. Introduction

As the human lifespan increases, the incidence of various spinal disorders is also increasing, leading to advancements in surgical techniques to address these disorders [1,2,3]. Among various spinal disorders, upper cervical disease is relatively rare. However, it carries high risks due to its anatomical structure. Surgical treatment has been limited for upper cervical disease, with non-invasive methods such as bracing and anterior surgical approaches being more commonly employed. However, braces are often uncomfortable for patients. In addition, they have a very low success rate [4], while anterior surgical approaches are associated with high perioperative morbidity [5].

Various techniques, such as Harms’ technique [6] and Yeom’s technique [7] have been reported, with posterior fusion emerging as a safe and effective treatment for upper cervical disease. In particular, precise and robust fixation of the C1-2 vertebrae has demonstrated satisfactory postoperative outcomes, even without extending fixation to the occiput, which has been previously associated with lower patient satisfaction [8]. Although atlantoaxial disease is rare, with surgeries not frequently performed, the critical location of this lesion often necessitates surgical intervention. Given the dramatic efficacy of surgical treatment, research on this topic is increasing.

Previous studies have primarily focused on symptom improvement and the stability of techniques following C1-2 posterior fusion, showing generally consistent results [9,10]. Although the C1-2 region experiences lower biomechanical loads than the lower cervical or thoracolumbar spine, it is closely related to the unique function of gazing. C2-3, the segment immediately adjacent to C1-2, is anatomically stable, but degenerative changes frequently occur at the C4-5-6 level postoperatively, which has the highest mobility in the cervical spine. The evaluation of postoperative changes was planned to be more quantitative and objective through radiological values. Therefore, this study aimed to analyze the risk factors associated with degenerative changes in the subaxial cervical spine following C1-2 surgery and examine related surgical parameters.

## 2. Materials and Methods

### 2.1. Population

This multicenter retrospective study was approved by the institutional review board, which waived the requirement for participant informed consent owing to the retrospective nature of the study, and utilized data from eight hospitals between January 2012 and December 2021. A total of 393 cohorts were identified using the prescription code for C1-2 posterior arthrodesis. Patients who underwent surgery that included the occiput or C3 and lower segments or who had a prior history of cervical spine surgery, fracture, infection, tumor, or congenital disease were excluded. Among the remaining cases, only those with successful bilateral C1-2 screw fixation and available clinical data, including X-rays up to approximately two years postoperatively, were selected. Finally, a total of 58 patients were included in this study. The surgical parameters analyzed included the method of screw fixation, fusion technique, decompression method, and complications observed over a two-year postoperative period.

Data collected included demographics, body mass index (BMI), history of rheumatoid arthritis (RA) and diabetes mellitus (DM), American Society of Anesthesiologists (ASA) score, length of hospital stay (LoS), operation time, and final follow-up duration.

### 2.2. Clinical and Radiologic Measurements

Neck pain was evaluated preoperatively, at three months postoperatively, and at two years postoperatively using the visual analog scale (VAS) via chart reviews. Radiologic parameters at these time points were assessed in the standing position using an electric caliper by two orthopedic surgeons (C.W. Bang and K.H. Kwon). Inter-observer agreement rates were calculated to minimize measurement errors, with agreement assessed using intraclass correlation (ICC) (ICC less than 0.5: poor reliability; ICC between 0.5 and 0.75: moderate reliability; ICC between 0.75 and 0.9: good reliability; ICC greater than 0.9: excellent reliability) [11] and Kappa coefficient (less than 0.2: poor agreement; 0.2–0.4: fair agreement; 0.41–0.6: moderate agreement; 0.61–0.8: substantial agreement; greater than 0.8: great agreement) [12]. The following parameters were evaluated:

► C1-2 Cobb angle (CA): The angle between the line that connects the middle of the anterior and posterior arch of C1 and the lower endplate of C2,
► C1-7 CA: The angle between the line that connects the middle of the anterior and posterior arch of C1 and the lower endplate of C7.
► T1 slope: The angle between the superior endplate of the T1 vertebra and a horizontal line.
► C1-7 sagittal vertical axis (SVA): Horizontal distance between the posterosuperior corner of the C7 vertebral body and a plumb line drawn from the anterior tubercle of C1.
► C2-7 SVA: Horizontal distance between the posterosuperior corner of the C7 vertebral body and a plumb line drawn from the centroid of C2.
► C4-6 dynamic angle: Difference between flexion CA and extension at the C4-6 segment.
► Listhesis distance at C4-5 (and C5-6): Difference between perpendicular linear distances from the superior extension line of C5 (C6) posterior wall to the inferior corner of the C4 (C5) body on flexion and extension.
► University of California at Los Angeles (UCLA) grade [13] and Pfirrmann grade [14] for assessing degeneration: Pfirrmann grade was measured preoperatively using MRI, as most patients lacked postoperative MRIs.

All radiologic parameters demonstrated a level of agreement corresponding to either ‘excellent’ or ‘great agreement’.

### 2.3. Definition of Radiologic Subaxial Pathology (RSP)

**Figure 1:**
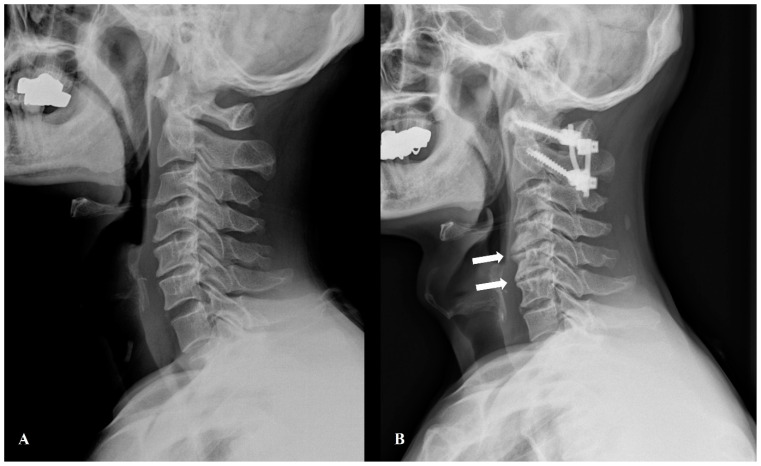
Preoperative plain radiograph (**A**) and radiologic subaxial pathology (white arrows) on postoperative plain radiograph (**B**).

A key element of this study was evaluating several radiologic indicators of spinal instability or arthritic changes. The difference in values between three months and two years postoperatively was calculated. Patients meeting at least two of the following five criteria were classified as having a “Radiologic Subaxial Pathology” (Figure 1):

► Increase of more than 5° in C4-6 dynamic angle
► Increase in listhesis distance at C4-5
► Increase in UCLA grade at C4-5
► Increase in listhesis distance at C5-6
► Increase in UCLA grade at C5-6

### 2.4. Statistical Analysis

All descriptive and comparative statistical analyses were conducted using IBM SPSS Statistics version 24 (IBM Corp, Armonk, NY, USA). The significance level was set at *p* < 0.05. Categorical variables were analyzed using the Pearson χ^2^ test or Fisher’s exact test as appropriate. Continuous variables were analyzed using Student’s *t*-test or the Mann–Whitney U test for non-normally distributed variables. Variables associated with RSP were identified as significant prognostic factors through univariate analysis (*p* < 0.05). Logistic regression was then performed to analyze prognostic factors.

## 3. Results

For screw fixation, lateral mass screws were used in approximately 84.4% of cases for C1. For C2, pedicle screws (62.0%) were the most frequently utilized, followed by mixed screws (29.3%), which combined pedicle screws, pars screws, and laminar screws. Regarding fusion methods, intra-articular fusion of the C1-2 facet joint with autobones or allobones was performed only in specific cases, such as occipital neuralgia, but was not conducted in the majority (86.2%) of patients. Posterior fusion using autobones from the posterior superior iliac spine was the most common (63.7%), while allobone fusion using demineralized bone matrix (DBM) was performed in 18.9% of cases. Decompression was rarely performed. The most common decompressive procedure was C1 arch removal, accounting for approximately 13.7% of cases. One case of vertebral artery injury occurred during surgery. However, the procedure was completed safely using compression and hemostatic agents. Postoperative complications included one case of low-grade infection requiring incision and drainage two weeks postoperatively and one case of rod breakage requiring rod replacement at postoperative 15 months. Mortality was not observed within three months postoperatively (Table 1).

When patients were divided into two groups based on the occurrence of RSP, the RSP group showed a significantly higher proportion of females (RSP: 69.2% vs. non-RSP: 50.0%, *p* = 0.019) and a higher prevalence of RA (RSP: 50.0% vs. non-RSP: 16.7%, *p* = 0.009). Other basic characteristics did not differ significantly between the two groups (Table 2). Preoperative neck pain and radiologic parameters were also similar between the RSP and non-RSP groups (Table 3). Postoperatively, neck pain at both 3 months and 24 months showed no significant difference between the two groups. However, at 3 months postoperatively, C1-7 SVA (RSP: 18.49 ± 14.63 mm vs. non-RSP: 26.87 ± 16.54 mm, *p* = 0.046) was significantly lower in the RSP group (Table 4). Multivariate regression analysis using significant variables (*p* < 0.05) such as sex, RA and 3-month C1-7 SVA in univariate analysis showed that RA (RR: 7.275; 95% CI: 1.680–31.497, *p =* 0.008) and 3-month C1-7 SVA (RR: 0.949; 95% CI: 0.908–0.993, *p* = 0.024) were significantly associated with RSP (Table 5). Given the close association between RA and C1-2 disease, additional analysis of basic characteristics was performed. Results revealed that patients with RA undergoing C1-2 surgery were younger (RA: 49.57 ± 13.82 years vs. non-RA: 61.78 ± 15.31 years). However, no other significant differences were found between RA and non-RA groups (Table 6).

**Figure 2:**
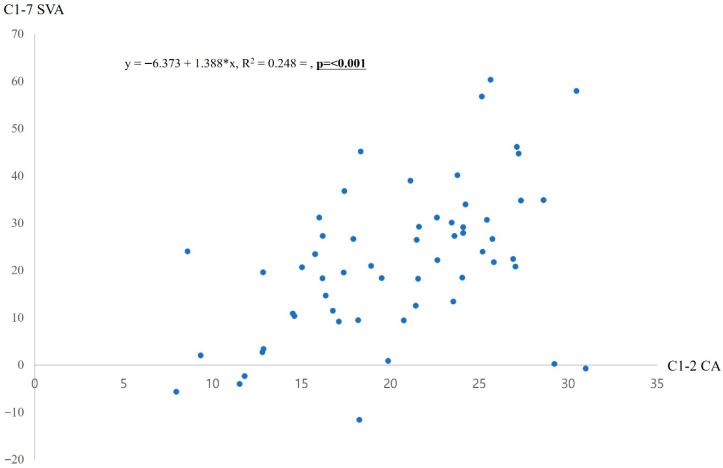
Linear regression analysis between C1-7 SVA and C1-2 CA. SVA, sagittal vertical axis; CA, Cobb angle. Asterisk (*) means multiple.

Among radiologic parameters related to surgery, 3-month C1-7 SVA was identified as the sole risk factor for RSP in multivariate analysis. To explore its correlations with other radiologic parameters at 3 months postoperatively, linear logistic regression analysis was conducted. Results showed significant positive correlations of 3-month C1-7 SVA with C1-2 Cobb angle (R^2^ = 0.248, *p* < 0.001) (Figure 2).

## 4. Discussion

Studies on upper cervical fusion have primarily focused on clinical symptoms, often using concepts derived from the more common lower cervical fusion or thoracolumbar fusion. However, the occipitocervical level that is preserved during C1-2 fusion has distinct pathophysiological characteristics due to its position at the top of the spinal column, its relatively low load from the brain and skull, and its complex functional demands, such as precise gaze control. This makes radiological evaluation significant, even before the onset of symptoms. Such evaluation provides a basis for examining degenerative changes at adjacent levels.

In spinal fusion surgeries, degenerative changes at adjacent levels should be crucially considered. This study analyzed radiological factors in subaxial cervical lesions because prior research has found that this level is most vulnerable to additional damage post-surgery, with the highest propensity for degenerative changes over time [15,16,17]. Radiological factors were specifically analyzed in this study, as they could help us assess the risk of adjacent segment degenerative changes, a common concern in spinal fusion surgeries. They could also guide postoperative management to better protect joints. Future studies incorporating longer-term cohorts and clinical adjacent segment pathology (ASP) correlations could enhance this research.

Previous studies, such as a study by Etebar and Cahill [18] have shown that female patients are more likely to develop postoperative adjacent segment disease after a spinal surgery than male patients. This study also observed a higher incidence of RSP in females in univariate analysis, potentially due to factors such as lower neck muscle mass and postmenopausal metabolic changes. However, when multivariate analysis was performed with other factors, gender was not a significant risk factor for the occurrence of RSP, which can be interpreted to mean that there may be factors with a higher risk than gender itself. Rheumatoid arthritis (RA) was also identified as a risk factor for RSP. While RA’s complexity, including its treatment modalities such as steroids, complicates its analysis, the findings of this study aligned with those of existing research linking RA to poor orthopedic and spinal surgery outcomes [19,20,21]. This study found minimal differences in baseline characteristics between RA and non-RA patients aside from a younger surgery age in the RA group, supporting the quality of data and addressing biases related to RA-induced secondary damage.

While cervical sagittal alignment has been extensively studied, it is less established than pelvic parameters at the lumbosacral level [22,23,24]. This is due to the cervical spine’s broad range of flexion–extension, rotational movement, a close relationship with horizontal gaze, and its integration with thoracic and lumbosacral structures. This study found that at three months after surgery, C1-7 SVA was the only radiological parameter significantly associated with subaxial cervical spine degenerative changes. Although prior studies have suggested that maintaining C2-7 SVA within 4 cm is ideal [25,26], differences within this range lack comprehensive research. We hypothesize that the flexion–compression force applied during C1-2 posterior fusion, which often positions the center of gravity [27] of the head posteriorly, may create subtle biomechanical changes in the cervical spine, potentially influencing degeneration if SVA approaches either extremely large or small values. Although C1-2 CA (RSP: 19.79 ± 6.00 vs. non-RSP: 21.29 ± 5.21, *p* = 0.327), C1-7 CA (RSP: 30.54 ± 9.44 vs. non-RSP: 34.28 ± 10.32, *p* = 0.158), and T1 slope (RSP: 19.01 ± 7.02 vs. non-RSP: 19.97 ± 5.95, *p* = 0.587) did not show statistically significant differences in this study, a trend of larger lordosis correlated with fewer degenerative changes was found, consistent with prior research studies [28,29]. In univariate analyses, these parameters, except SVA, did not show direct or significant associations with RSP occurrence (*p*-value range: 0.158–0.587). However, linear regression analysis revealed significant positive correlations between C1-2 CA and SVA, suggesting that larger C1-2 CA could indirectly influence RSP occurrence by optimizing SVA. The authors speculate that individual values such as C1-2 are not directly related to RSP, but are related to SVA, which is directly related to RSP, and thus C1-2 may be indirectly related to RSP.

The UCLA grade and Pfirrmann grade of C4-6 were preoperative factors predicted to have strong associations with radiologic subaxial pathology. These factors are well-documented risk factors for adjacent segment degenerative changes following fusion surgeries in other spinal regions, such as the cervical or lumbosacral spine [30]. However, in this study, they showed no statistically significant correlations with radiologic subaxial pathology (Table 3). This finding could be interpreted as follows. First, this study focused on radiologic subaxial pathology rather than clinical ASP, aiming to achieve early detection of disease and determine progression. Since UCLA and Pfirrmann grades are more distinct in advanced stages of the disease (e.g., grade 3 or higher) rather than in early-stage lesions such as grade 1 or 2, they might have limitations in predicting the radiologic pathology targeted in this study. Second, for cases with a grade 3 or higher disease representing more advanced stages, joints may exhibit significant instability, potentially progressing to joint destruction, although these joints might stabilize in some instances. This was supported by the observation in this study that dynamic angles and listhesis distances tended to stabilize in cases with higher UCLA and Pfirrmann grades. To address this loophole, the authors integrated multiple factors to comprehensively define RSP, thereby compensating for the shortcomings of these two grading systems.

This study has several limitations besides its retrospective design and small sample size, resulting in low power of the statistical analysis. First, because data were collected from eight hospitals belonging to the same university, there might be issues with the accuracy of factors such as surgical procedures and clinical symptoms recorded. To address this, the authors only enrolled 58 patients from a cohort of 393, focusing on those whose medical records were well-preserved. For surgical procedures, post-operative plain radiographs and surgical records were cross-checked and analyzed, which allowed for some degree of correction. Second, the definition of RSP might be an issue. Since CT and MRI scans were not routinely performed post-surgery, only plain radiographs were used to assess degenerative changes, which could be a weakness. However, this study made the best use of available radiographs, with RSP defined based on the change before and after surgery, not absolute values. Thus, if the focus is on understanding the trend towards degenerative changes rather than diagnosing definitive degenerative changes, this limitation could be addressed to some extent. Thirdly, due to the cohort nature of this multicenter study, we were unable to analyze the types of medications used for rheumatic diseases. For the same reason, we were unable to differentiate between medications, such as bone density, smoking status, and steroid use. Additionally, because MRI or CT was often not performed and the study plan focused on initial assessment, analyses of long-term outcomes beyond 5 years were not performed. Therefore, we could not correlate the radiographic progression of degenerative change with clinical outcome scores and evaluate the union rate. These limitations need to be addressed in future studies.

## 5. Conclusions

C1-2 posterior fusion surgery is biomechanically distinct from other spinal segments. Identifying risk factors for subaxial level pathology following C1-2 fusion surgery is essential. This study identified RA as the most significant risk factor for subaxial level pathology following C1-2 fusion surgery. This could be interpreted as suggesting that patients undergoing C1-2 fusion surgery also have a similar association with the risk factors for existing cervical degenerative changes. Among various surgical factors, most of the radiologic parameters were not directly associated with subaxial level pathology, except for C1-7 SVA. Further research on the relations of cervical sagittal parameters with C1-7 SVA could help elucidate more direct correlations with surgical outcomes.

## Figures and Tables

**Table 1:** Surgical characteristics of the patients.

Variables			N (%)
Screw			
C1	Lateral mass screw		49(84.4%)
	Posterior arch screw		9 (15.5%)
C2	Pedicle screw		36 (62.0%)
	Pars screw		4 (6.8%)
	Laminar screw		1 (1.7%)
	Mixed screw		17 (29.3%)
Fusion			
Intra-articular	Autobone	Uni-lateral	1 (1.7%)
		Bi-lateral	2 (3.4%)
	Allobone	Uni-lateral	3 (5.1%)
		Bi-lateral	2 (3.4%)
	None		50 (86.2%)
Posterior	Autobone		37 (63.7%)
	Allobone		11 (18.9%)
	Autobone + allobone		3 (5.1%)
	None		7 (12.0%)
Decompression			
C1			8 (13.7%)
C2			3 (5.1%)
C1 + C2			3 (5.1%)
Complication			
Vertebral a. injury			1 (1.7%)
Revision surgery			2 (3.4%)
3 months mortality			0

**Table 2:** Basic characteristics of patients with or without a radiologic subaxial pathology.

	RSP (*n* = 34)	%	Non RSP (*n* = 24)	%	*p*
Sex					0.019 *
Male	7	32.8	12	50.0	
Female	27	69.2	12	50.0	
Age (years)	55.91 ± 15.53		59.42 ± 16.31		0.411
BMI (kg/m^2^)	24.22 ± 3.24		24.26 ± 3.49		0.962
RA	17	50.0	4	16.7	0.009 *
DM	10	29.4	7	29.2	0.984
ASA					0.289
1	9	26.5	4	16.7	
2	23	67.6	20	83.3	
3	2	5.9	0	0	
LoS (days)	18.91 ± 15.82		19.46 ± 15.77		0.897
Operation time (min)	281.65 ± 75.43		277.33 ± 69.48		0.826
Follow-up period (days)	869.88 ± 208.44		932.13 ± 285.90		0.341

Data are presented as means ± standard deviations. RSP, radiologic subaxial pathology; BMI, body mass index; RA, rheumatoid arthritis; DM, diabetes mellitus; ASA, American Society of Anesthesiologists; LoS, length of stay in hospital. * *p* < 0.05, statistical significance.

**Table 3:** Preoperative clinical outcomes and radiologic parameters of patients with or without a radiologic subaxial pathology.

	RSP (*n* = 34)	Non RSP (*n* = 24)	*p*
VAS	4.00 ± 2.71	4.42 ± 3.12	0.591
C1-2 CA (°)	22.68 ± 10.08	20.87 ± 8.56	0.476
C1-7 CA (°)	33.42 ± 11.63	33.38 ± 9.23	0.988
T1 slope (°)	19.68 ± 5.67	20.61 ± 8.15	0.608
C1-7 SVA (mm)	20.10 ± 14.01	24.24 ± 16.29	0.305
C2-7 SVA (mm)	12.65 ± 13.94	15.04 ± 16.49	0.553
C4-6 dynamic CA (°)	24.87 ± 8.95	21.61 ± 9.94	0.198
C4-5			
Listhesis (mm)	1.12 ± 0.75	1.16 ± 1.11	0.854
ULCA			0.802
Gr ≤ 2	65.7%	68.8%	
Gr ≥ 3	34.3%	31.3%	
Pfirrmann			0.389
Gr ≤ 3	73.3%	60.0%	
Gr ≥ 4	26.7%	40.0%	
C5-6			
Listhesis (mm)	0.86 ± 0.71	0.57 ± 0.78	0.151
UCLA			0.464
Gr ≤ 2	51.4%	40.6%	
Gr ≥ 3	48.6%	59.4%	
Pfirrmann			0.425
Gr ≤ 3	63.3%	52.0%	
Gr ≥ 4	36.7%	48.0%	

Data are presented as means ± standard deviations. RSP, radiologic subaxial pathology; VAS, visual analog scale; CA, Cobb angle; SVA, sagittal vertical axis; UCLA, University of California at Los Angeles grading.

**Table 4:** Postoperative clinical outcomes and radiologic parameters of patients with or without a radiologic subaxial pathology.

	RSP (*n* = 34)	Non RSP (*n* = 24)	*p*
VAS			
3 month	0.85 ± 1.04	1.63 ± 2.06	0.101
24 month	1.32 ± 1.27	1.88 ± 1.89	0.222
**Postoperative**			
C1-2 CA (°)	19.79 ± 6.00	21.29 ± 5.21	0.327
C1-7 CA (°)	30.54 ± 9.44	34.28 ± 10.32	0.158
T1 slope (°)	19.01 ± 7.02	19.97 ± 5.95	0.587
C1-7 SVA (mm)	18.49 ± 14.63	26.87 ± 16.54	0.046 *
C2-7 SVA (mm)	10.45 ± 12.59	15.79 ± 11.88	0.109
C4-6 dynamic CA (°)	22.18 ± 9.28	23.06 ± 11.37	0.749
C4-5 listhesis (mm)	1.41 ± 0.97	1.61 ± 1.27	0.503
C5-6 listhesis (mm)	1.12 ± 0.89	0.86 ± 0.99	0.301

Data are presented as means ± standard deviations. RSP, radiologic subaxial pathology; VAS, visual analog scale; CA, Cobb angle; SVA, sagittal vertical axis. * *p* < 0.05, statistical significance.

**Table 5:** Multivariate regression analysis of the initial signs of osteoarthritis.

Variables	Multivariate Relative Risk (95% CI)	*p*
RA	7.275 (1.680–31.497)	0.008 *
3 months C1-7 SVA	0.949 (0.908–0.993)	0.024 *

CI, confidence interval; RA, rheumatoid arthritis; SVA, sagittal vertical axis. * *p* < 0.05, statistical significance.

**Table 6:** Basic characteristics of patients in the rheumatoid and non-rheumatoid cohorts.

	RA (*n* = 21)	%	Non RA (*n* = 37)	%	*p*
Sex					0.274
Male	5	23.8	14	37.8	
Female	16	76.2	23	62.2	
Age (years)	49.57 ± 13.82		61.78 ± 15.31		0.004 *
BMI (kg/m^2^)	23.13 ± 3.34		24.86 ± 3.18		0.055
DM	8	38.1%	9	24.3%	0.268
ASA					0.840
1	4	19.0%	9	24.3%	
2	16	76.2%	27	73.0%	
3	1	4.8%	1	2.7%	
LoS (days)	20.48 ± 17.20		18.38 ± 14.91		0.628
Operation time (min.)	281.67 ± 63.36		278.84 ± 77.93		0.888
Follow-up period (days)	875.05 ± 231.63		907.32 ± 251.73		0.634

Data are presented as means ± standard deviations. RA, rheumatoid arthritis; BMI, body mass index; DM, diabetes mellitus; ASA, American Society of Anesthesiologists; LoS, length of stay in hospital. * *p* < 0.05, statistical significance.

## Data Availability

The original contributions presented in this study are included in the article. Further inquiries can be directed to the corresponding author.
